# Giant Plexiform Schwannoma of the Tongue

**DOI:** 10.1155/2011/762524

**Published:** 2011-11-24

**Authors:** Lluís Nisa, Toni von Büren, Amine Tiab, Roland Giger

**Affiliations:** ^1^Department of ENT., Hôpital de Sion-CHCVs-RSV, Avenue du Grand Champsec 80, 1950 Sion, Switzerland; ^2^Department of Pathology, Central Institute of the Valais Hospitals, 1950 Sion, Switzerland

## Abstract

We present the case of a 38-year-old woman that presented with a very slowly enlarging mass of the tongue. MRI revealed a large mass originating in the tongue base and extending to the supraglottic space. Biopsy of this tumor confirmed the diagnosis of schwannoma. An endaural approach allowed complete resection of the tumor. Definitive histopathological examination showed a plexiform schwannoma. Schwannoma or neurilemoma represents a benign tumor potentially affecting any nerve. The head and neck region is a relatively common location for schwannomas, but tongue schwannomas are considered to be rare and pose the problem of both clinical and histological differential diagnosis of tongue masses.

## 1. Introduction

Schwannoma or neurilemoma is a benign tumor originating from the Schwann cell of the nerve sheath. It usually presents as an individual and encapsulated tumor and can virtually affect any peripheral, autonomic, or cranial nerve. Plexiform schwannoma is an anatomical variant of schwannoma characterised by intraneural and multinodular growth, most often involving the skin and subcutaneous tissues of the head and neck region [1]. Twenty-five to forty-five percent of all schwannomas are considered to affect the head and neck region, and one percent are intraoral [2].

We report the case of a patient presenting a giant schwannoma of the tongue base, presenting as a slowly enlarging mass. Publication of this case was approved by the Ethical Committee of our institution.

## 2. Case Report

**Figure 1:**
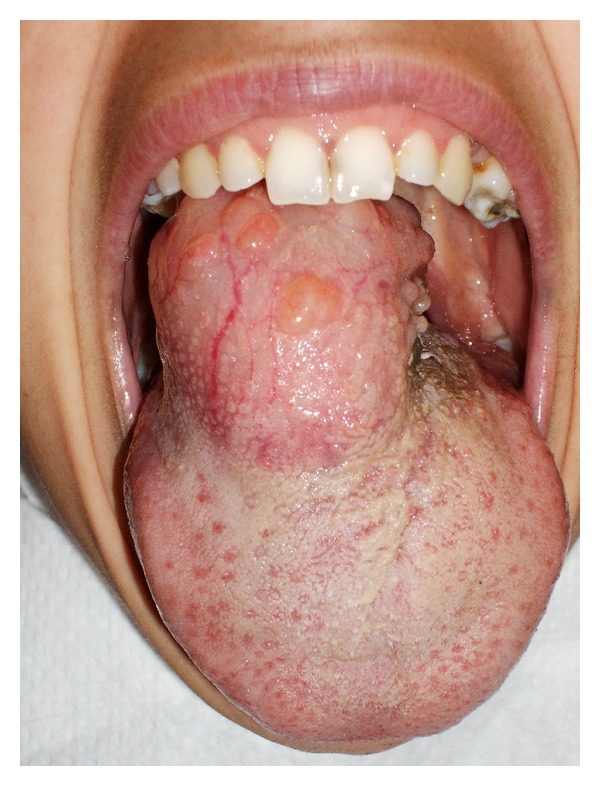
Oral cavity examination showing a large mass of the tongue base.

A 38-year-old Algerian woman in previous good health, presented with a twenty-year history of a slowly enlarging mass of the tongue. She sought for an ENT consultation at our hospital because of progressive dyspnoea, dysphonia, and severe dysphagia. Further interrogation revealed a very important asthenia (to the point that she had to stop working), involuntary loss of 10 kg in one year (she could only swallow liquids), and a family history of hypothyroidism. Oral cavity examination revealed a large, mobile, and hard mass of the tongue base, obstructing almost completely the oropharynx and part of the supraglottic area (Figure 1).

We suspected an ectopic thyroid in the first place and performed a neck ultrasound that showed a thyroid gland in normal position. Doppler analysis suggested thyroiditis. Laboratory workup revealed hypothyroidism (high TSH, low T4 and T3) and a high title of antithryoid peroxidase antibodies (anti-TPO), confirming Hashimoto disease.

**Figure 2:**
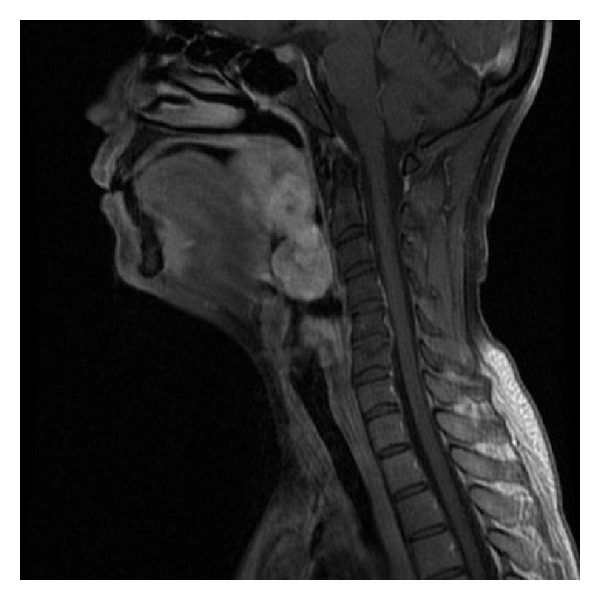
Magnetic resonance imaging of the head and neck assessing the deep extension of the tongue mass.

Magnetic resonance imaging (MRI) showed an 8.5 × 5 × 6 cm pedicled bilobed basilingual mass, well delimited and encapsulated (Figure 2).

A highly cellular tissue organised in palisading fascicles, S100-positive cells, and rare atypical cells was seen at immunohistochemical examination of a biopsy of the mass, suggesting the diagnosis of schwannoma.

Substitution therapy for Hashimoto disease was started immediately.

**Figure 3:**
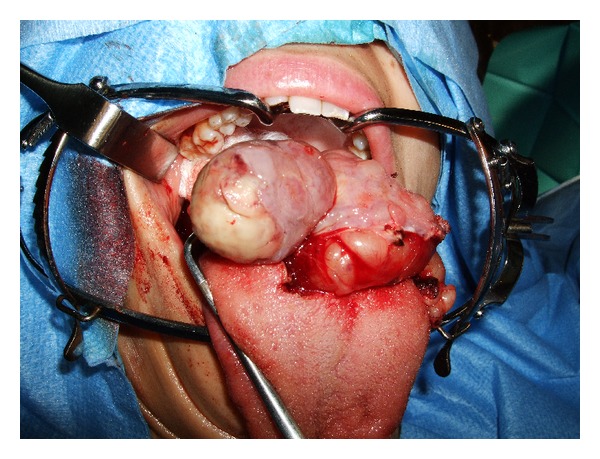
Per-operative aspect of the tongue schwannoma.

Surgical removal of the tumor was decided. After induction, nasotracheal intubation was guided with a fiberoptic bronchoscope passed through the tube. We then performed an endoral excision of the mass, which presented a well-delineated plan separating it from the surrounding tongue muscles (Figure 3). Cold-steel dissection allowed undermining the whole volume of the tumor, and only a 2 × 1 cm patch of lingual mucosa was removed with the tumor. Primary closure of the tongue muscles and mucosa was performed.

**Figure 4:**
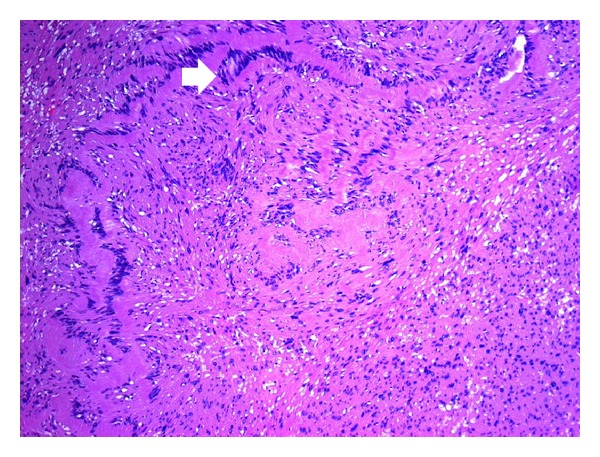
H&E stain (×20) of the plexiform schwannoma, displaying the typical palisading organisation (white arrow).

No primary nerve originating this lesion was macroscopically identified during surgery. The tongue was closed by primam intentionem and the patient was immediately extubated, with an uneventful postoperative course. Definitive histopathological analysis confirmed the diagnosis of plexiform schwannoma of the tongue (Figure 4).

## 3. Discussion

The tongue is the most common location for intraoral schwannoma, followed by the palate and the oral mucosa. Tongue schwannoma shows no gender predilection and may present at any age (especially in the third decade of life). Often appearing as a painless and slowly enlarging mass of the tongue, when schwannomas reach a certain size they may cause dysphagia, voice changes, and breathing difficulties [3].

Our patient had a family history of hypothyroidism, which lead us to suspect lingual thyroid. This was ruled out by ultrasonographic examination showing a morphologically normal thyroid gland in its usual basicervical position. The diagnosis of Hashimoto disease would at least partially explain the asthenia, but since there is not an established relationship between tongue schwannoma and autoimmune thyroiditis, the association of these entities in our patient is probably coincidental.

Entities such as lipoma, hemangioma, eosinophilic granuloma, epidermoid and dermoid cysts, epithelial hyperplasia, granular cell tumor, benign salivary gland tumors, rhabdomyoma, leiomyoma, lingual thyroid, and lymphangioma, are all benign soft-tissue lesions that should be considered, in addition to schwannoma, in the differential diagnosis of a slowly growing and macroscopically circumscribed mass of the tongue. In some cases, glandular malignant processes, squamous cell carcinoma and sarcomas may share similar clinical features [4, 5].

Peripheral nerve sheath tumors can be benign or malignant. Benign peripheral nerve sheath tumors include basically schwannoma and neurofibroma, the latter usually found in individuals with neurofibromatosis type 1. Malignant peripheral nerve sheath tumors are a rare variety of soft-tissue sarcoma, presenting very rarely in the head and neck region and carrying poor prognosis [6].

Immunohistochemical examination should allow distinguishing schwannomas from other nerve-originating tumors. Two different histopathological schwannoma patterns are recognised. Antoni A, with high cellularity and little stromal matrix is the commonest form, and Antoni B, with lower cellular growth and a myxoid stroma. Plexiform schwannomas share the basic features of other schwannomas, especially its benign nature with no metastatic potential, but the distinction between plexiform schwannoma and plexiform neurofibroma or even malignant peripheral nerve sheath tumours can be more difficult [1, 7]. Several members of the S100 protein family, which are normally present in cells derived from the neural crest (Schwann cells, melanocytes and glial cells), are used as immunohistochemical markers for certain skin and nerve sheath tumours. S100 proteins are currently the most sensitive marker for tumors with schwannian differentiation, and the study of differential expression and distribution of certain S100 subtypes may be a tool contributing to distinguish between benign and malignant peripheral nerve sheath tumours [8].

Radiological exams permit to assess the extension of the lesion, and they are useful as preoperative workup. Magnetic resonance imaging is the best exam to show deep lesions of oral cavity soft tissues. Even if a certain number of particular features are found in the imaging of schwannomas, none is specific. An overlap exists in the imaging characteristics of schwannoma and other soft-tissue tumors, especially neurofibroma [9].

Treatment of schwannomas of the tongue is surgical. A transoral approach allows us to completely remove even the largest lesions, with a very low rate of morbidity and recurrency [3].

Our patient's tumor measured 85 mm in greatest size, becoming the largest schwannoma of the tongue reported so far in the literature to our knowledge.
